# The Effects of Copper Constituent of Coin Currency on Embryonic Zebrafish Development

**DOI:** 10.1155/2021/2134928

**Published:** 2021-02-04

**Authors:** Ted Inpyo Hong, Seo-Gu Kang, Yu-Ri Lee, Tae-Ik Choi, Woo-Keun Kim, Cheol-Hee Kim

**Affiliations:** ^1^Department of Biology, Chungnam National University, Daejeon 34134, Republic of Korea; ^2^System Toxicology Research Center, Korea Institute of Toxicology, Daejeon, Republic of Korea

## Abstract

Copper has demonstrated utility in multiple industrial applications for its high conductivity and antibacterial/antiviral properties. However, numerous findings have suggested potential hazards regarding pathogenesis. This study was conducted to demonstrate the application of zebrafish (*Danio rerio*) as a cost-effective biological assay to detect environmental pollution, i.e., heavy metal of coins. We demonstrated that zebrafish larvae exposed to copper-plated coins or copper (II) ion solution elicited a consistent phenotype of early mortality without signs of morphological defects in surviving individuals. Copper ion solution served as a standard to (1) corroborate copper exposure from coins and (2) demonstrate proportional increase in early mortality phenotype according to concentration. We found that 5 *μ*M CuSO_4_·5H_2_O was the minimal concentration to elicit the observed phenotypes from copper toxicity. This study aimed to demonstrate how a simple protocol involving wild-type zebrafish larvae could provide an economical solution to water monitoring in areas of rapid technological advancement and increasing environmental concerns, especially in communities without access to expensive analytical methods.

## 1. Introduction

With the steady and rapid rise of the global electrical and electronic equipment (EEE) market, heavy metal pollution is coming to the forefront as a major environmental issue [1]. Unlike organic contaminants, heavy metals are in effect nonbiodegradable, thus tending to persist and accumulate in affected habitats and biotas [2]. In particular, the contamination of water sources is of particular concern to humans [3]. Studies have purported that water contamination is the leading cause of morbidity and mortality worldwide [4]. The disruption of finely tuned homeostatic balance of trace essential elements, such as copper, can give rise to serious health consequences.

The US Environmental Protection Agency classified copper as an antimicrobial agent in 2008 and limited acceptable levels of copper to 1.3 mg/L in drinking water [5]. However, studies, such as one by Sparks and Schreurs, have reported that, even at low concentrations (0.12 mg/L), copper added to the drinking water of rabbits could lead to learning deficits and induction of amyloid-*β* plaques in the brain (as those seen in Alzheimer's disease patients) [6]. It follows that regular surveillance of aquatic resources is an important step to ensure the safety of water quality; however, current analytical methods such as high-performance liquid chromatography (HPLC), although providing precision and accuracy, are generally cost-intensive, complex, and inaccessible for the majority of underdeveloped communities. Low-tech options such as test strips or digital tools, although being more accessible, are either too target-specific or may still be too complex/expensive for wide application. Our study demonstrates how normal (wild-type) zebrafish larvae can be implemented as a means of water quality assessment based simply on counting the number of expired larvae. Such a biological assay could serve as an effective preliminary measure to assess quality of water in developing communities.

As an aquatic animal model, zebrafish are highly suited for large-scale toxicological studies due to rapid generation time, transparency of the developing larvae, and ease of administration of test compounds. We chose to focus on heavy metals in this study given the growing problem of heavy metal contamination in increasingly industrializing countries of developing regions of the world [7, 8]. In particular, we focused on copper metal because of (1) its ubiquity and (2) in order to establish a reference standard using copper (II) ion solution and bypass hi-tech analytical methods. While wild-type zebrafish could serve as an ecologically safe (i.e., nongenetically modified) preliminary measure for water quality assessment, its application to other contaminant targets, such as endocrine disrupters and organic pollutants [9], demonstrates their adaptability to more precise analyses down the line. For example, areas known for high levels of specific metal or organic contaminants could directly employ zebrafish modified with aromatic hydrocarbon response elements (AHREs) or metal response elements (MREs), utilizing easily quantifiable fluorescent reporters [10, 11]. Nontransgenic options include metal-specific chemosensors which can equip wild-type zebrafish as water quality indicators measured by fluorescence intensity [12].

In this study, we found premature death as the main phenotype of zebrafish larvae exposed to copper-plated coins or copper (II) ion solution. Of note, no morphological defects were observed among surviving individuals in the stages assessed in the study. Copper toxicity-related phenotypes, i.e., mortality rate, increased with stepwise elevation of copper (II) ion concentration, ranging from 0.5 nM to 400 *μ*M, when using copper sulfate pentahydrate as a chemical standard. We demonstrated the use of wild-type zebrafish as a biological assay for water assessment, which illustrates a potential application toward improving water quality standards in developing regions.

## 2. Materials and Methods

### 2.1. Animal Care

Embryos used in this study were obtained from matings of wild-type zebrafish from the Zebrafish Center for Disease Modeling (ZCDM; Daejeon, Republic of Korea). Fish were raised and maintained under standard conditions [13]. Prior to experiment, all embryos were placed in an incubator at 28.5°C. All embryos were kept in egg water from spawning and throughout the experiments. Egg water preparation consisted of sea salts (Sigma Life Sciences; St. Louis, USA) in triple distilled water. Experiments were conducted according to guidelines approved by the Animal Ethics Committee of Chungnam National University (CNU-00866).

### 2.2. Zebrafish Toxicity Bioassay

#### 2.2.1. General Procedure

Toxicity assays involved allocating early-stage WT zebrafish embryos to a 6-well cell culture plate (SPL Life Sciences, Pocheon-si, Republic of Korea). Treatment was added to certain wells according to the kind of toxicity assay being conducted, and the culture plate was kept in a 28.5°C culture chamber throughout the experiment.

#### 2.2.2. Assay Utilizing Various Currency Coins

For the toxicity assay using coins, coins from various countries of origin were obtained from general circulation currency. Selected coins included the following: a Singapore dollar (1 SGD), 5 Italian lira (5 ITL), a Canadian dime (0.1 CAD), a US penny (0.01 USD); and 500, 100, 50, and 10 South Korean won (500, 100, 50, 10 KRW). Composition profiles were obtained from various internet sources including respective monetary authorities. Prior to experiments, coins were sterilized by autoclave and their masses weighed. Zebrafish embryos of approximately sphere stage (~4 hours post fertilization or hpf) were allocated to sterile cell culture plates containing egg water and either one coin from the aforementioned list (no duplicates) or no coin, as control. Plates were incubated and assessed at 19 hours post exposure (hpe).

#### 2.2.3. Assay Utilizing Copper-Plated Pennies

For toxicity assays using US pennies, US general circulation pennies were mechanically cleaned and sterilized by autoclave. The experiment was conducted in triplicate. One replicate consisted of ten embryos loaded into a well of a 6-well cell culture plate with either no coin addition, as control, or addition of a US 1 cent piece added to the respective well at one of the following developmental stages: 1-cell stage (0.25 hpf), sphere stage (4 hpf), bud stage (10 hpf), and 10-somite stage (14 hpf). Assessments and imaging were performed at 12, 24, and 48 hpe. Representative images from the three replicates were selected for presentation in the figure. Due to technical difficulties, some developmental stages were not included in the assessment (i.e., 12 and 24 hpe assessment of “bud” and “10-somite” and 12 hpe for “sphere”).

#### 2.2.4. Assay Using Copper (II) Ion Solution

In toxicity assays using copper sulfate pentahydrate (CuSO_4_·5H_2_O, assay ≥ 99.0%; Samchun Pure Chemical Co., Republic of Korea) as a standard chemical, zebrafish embryos of approximately 64-cell stage (~2 hpf) were allocated to a 6-well cell culture plate, each treatment well containing either 400, 100, 25, or 5 *μ*M CuSO_4_·5H_2_O in egg water, along with an egg water control well. Subsequently, a serial dilution of lower copper sulfate concentration was conducted in which pregastrulation embryos (approximately 256-cell stage) were allocated to individual wells of a 6-well cell culture plate containing either egg water control or 5 *μ*M, 500 nM, 50 nM, 5 nM, or 0.5 nM CuSO_4_·5H_2_O solution.

### 2.3. Statistics and Data Analysis

Samples were assessed by quantification of mortality and imaging at the indicated time point of each respective experiment. Samples were imaged using either a Leica MZ16 or S6E stereo microscope (Leica Microsystems; Wetzlar, Germany). Percent mortality at each observation point was calculated and plotted using GraphPad Prism 5 (GraphPad Software; San Diego, USA).

## 3. Results and Discussion

**Figure 1:**
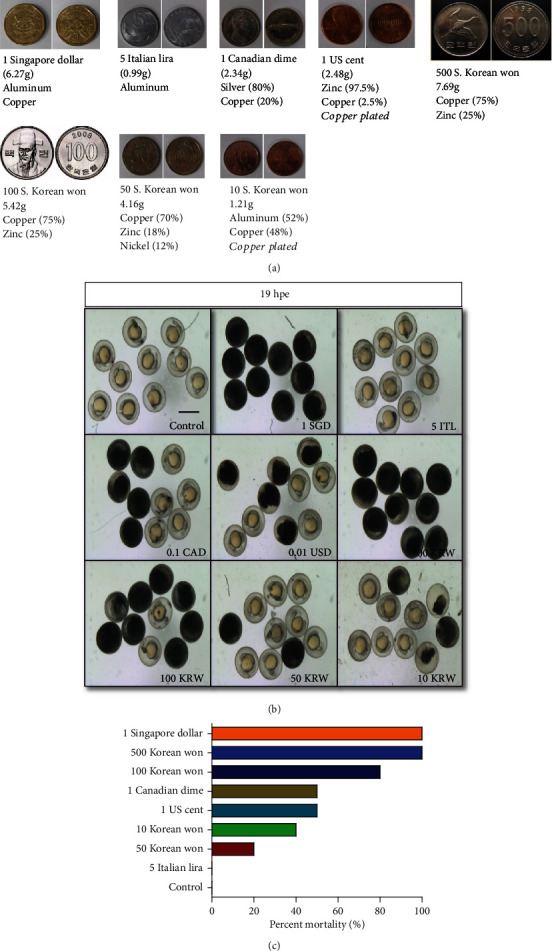
Effects of various coin currencies on developing zebrafish embryo. (a) Different coin currencies described by country of origin, monetary value, mass, and composition. (b) Comparison of embryo (*n* = 10) morphology at 19 hpe after addition of coins; hpe: hours post exposure; control: embryos in egg water (no addition); SGD: Singapore dollar; ITL: Italian lira; CAD: Canadian dollar; USD: US dollar; KRW: South Korean won. Scale bar: 200 *μ*m. (c) Quantification of early mortality at 19 hpe, according to group.

Although coin currency does not contribute to environmental heavy metal pollution, per se, the mining of metallic ore involved in coin production acts as a worldwide contributor [14]. We chose coins in our study due to (1) their ubiquity and (2) studies which have demonstrated coselection of antibiotic and metal resistance in bacterial communities exposed to leached metal in aquatic repositories for tossed “good luck” coins [15]. The coins used in this experiment had diverse metal composition (Figure 1(a)) and structure (e.g., alloy or metal plating) and showed different effects on larval mortality. Notably, all coins containing some proportion of copper metal exhibited toxic effects, while the only fully viable group was the all-aluminum Italian lira. At 19 hpe, early mortality was the most common effect on developing zebrafish embryos. The 1 SGD and 500 KRW coins resulted in complete mortality, while three other groups (100 KRW, 0.1 CAD, and 0.01 USD) demonstrated 50% or higher mortality rate (Figure 1(c)). Interestingly, developmental deformity or delay was not observed in surviving individuals, except for a few instances.

**Figure 2:**
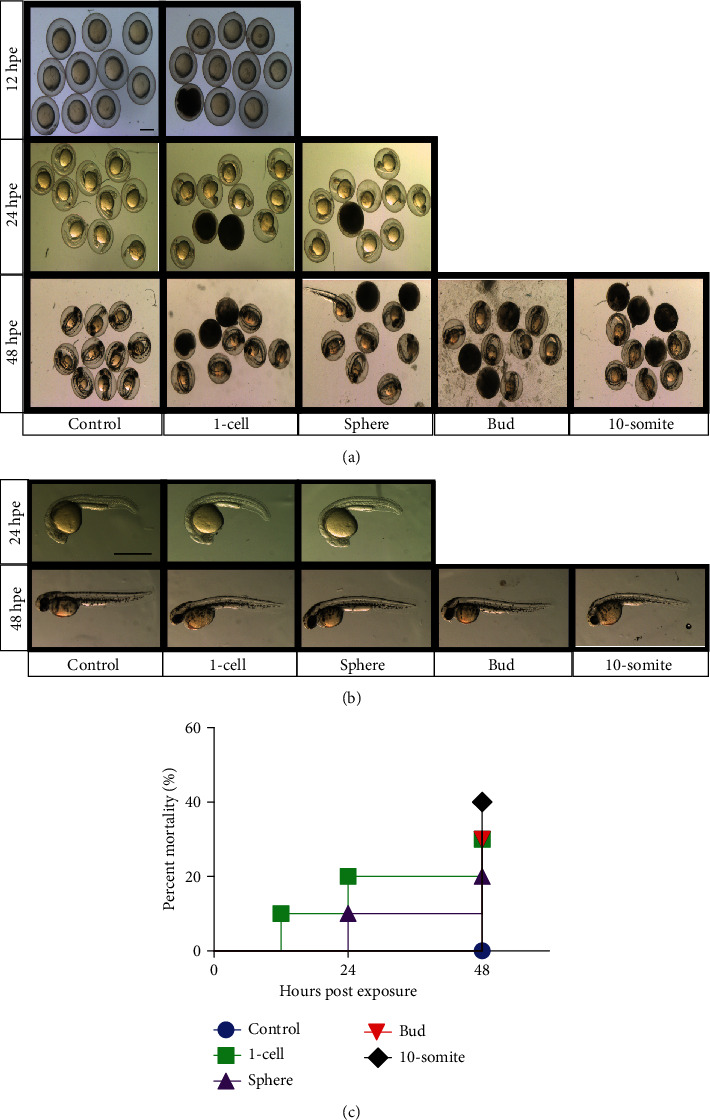
Assessment of effects by copper-plated coin. (a) Effects of US 1 cent coin on developing embryos (*n* = 10) at differential stages of development, indicating time point of exposure to penny: 1-cell (~0.25 hours post fertilization or hpf), sphere (~4 hpf), bud (~10 hpf), 10-somite (~14 hpf); control: embryos in egg water (no addition). (b) Magnification of representative image for relevant stages and time points. Scale bars: 200 *μ*m. (c) Survival curve showing percent mortality at indicated stages and time points.

We inferred copper leakage into the well medium based on previous evidence of metal leaching from coins in aqueous environments (Martinez et al., 2020), reasonable deduction from experimental design, such as control of the aqueous medium, and the experimental results. As previously mentioned, all copper-containing coin groups demonstrated some level of mortality, as the majority of groups (5 of 7) exhibited 50% or greater mortality. Following, we assessed the effects of a copper-only exposure (i.e., copper-plated coin) on embryo development according to different stages of exposure. Mortality rate generally increased from 24 to 48 hpe, while the developmental stage of coin addition did not appear to affect mortality (Figures 2(a) and 2(c)). At 48 hpe, coin addition during 1-cell stage showed similar mortality rate as that of bud stage, despite a 10 h advance in development. Embryos exposed to copper-plated coins showed no obvious morphological defects at 24 hpe; however, some stages (1-cell, sphere, and 10-somite) exhibited slight downward curvature of the body compared to wild-type, starting at 48 hpe (Figure 2(b)). Of note, most embryos were still encased in chorion before imaging at 48 hpe and could have been thusly influenced in shape.

**Figure 3:**
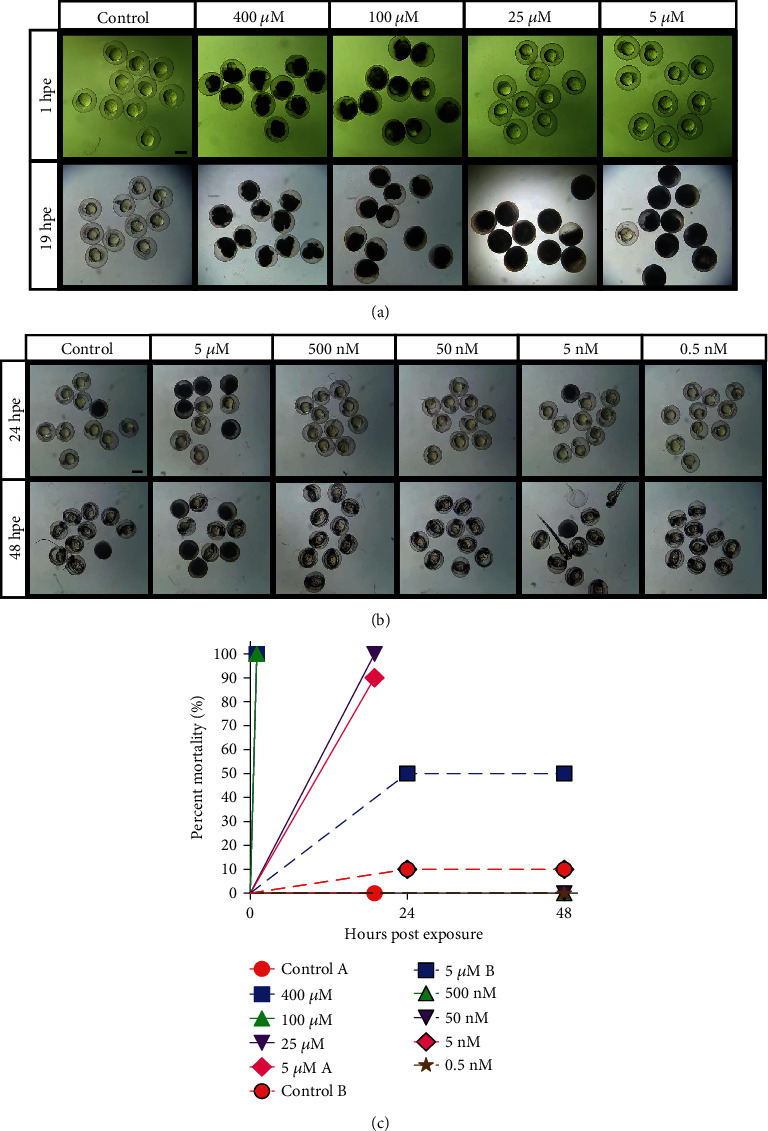
Effects of copper (II) ion solution on developing zebrafish embryos. (a) Higher concentration range of copper sulfate pentahydrate observed within 24 hours post exposure (hpe) at 1 and 19 hpe. (b) Lower concentration serial dilution of copper sulfate solution observed over a 48 h period at 24 h intervals. Control: embryos in egg water (no copper solution). Concentrations in header indicate the concentration of copper sulfate solution. Scale bars: 200 *μ*m. (c) Aggregate of mortality data quantified from (a) and (b). Repeat labels are differentiated by letter of respective panel.

The phenotypic similarity in experiments 1 and 2 (Figures 1 and 2) suggested leached copper ions as the main effector. In order to corroborate this inference, we repeated the toxicology assay using high-purity copper (II) ion solution as a standard chemical reference and examined the resulting effects on larvae. We found increasing levels of larvae mortality according to stepwise increase in copper sulfate concentration (400, 100, 25, and 5 *μ*M), leading to 90-100% mortality by 19 hpe (Figures 3(a) and 3(c)). Additional serial dilutions were performed starting from 5 *μ*M to 0.5 nM to examine lower concentrations. Over a 48 h period, 5 *μ*M copper sulfate exhibited 50% early mortality without any obvious signs of morphological defect, compared to control, while all concentrations within the nanomolar order of magnitude showed 0% mortality except for a single mortality in the 5 nM group (as well as one in the control group which may have been due to individual variability) (Figure 3(b)).

Given the heavy metal composition of coin currency, we were interested in the effects of coin exposure on the developing zebrafish embryo. Zebrafish embryos exposed to different coin currencies demonstrated a phenotype of early mortality with a general absence of early developmental abnormality. The zebrafish model animal has become popular in the study of human genetic diseases. Studies estimate that about 70% of human genes have at least one orthologue in zebrafish [16]. Moreover, multiple aspects of neurodevelopment including cell proliferation, differentiation, and connectivity are governed by related pathways between zebrafish and humans [17–19]. Our findings in this study may additionally suggest potential hazards to vulnerable groups, such as infants, from metal-based objects like coins. Indeed, studies have demonstrated risk of metal allergies from the handling of coins [20] and have even reported fatal cases from instances of massive coin ingestion [21].

## 4. Conclusions

Taken together, our study demonstrated the feasibility of utilizing zebrafish as a cost-effective tool for monitoring aquatic pollutants, such as copper, especially in developing regions undergoing rapid industrialization. The toxic effects of copper metal resulted in premature death in zebrafish larvae with the general absence of early morphological defects. Results showed onset of mortality phenotype starting at concentration of 5 *μ*M copper sulfate pentahydrate and stepwise increase with elevation of copper concentration. This study demonstrates a case in point of how a simple biological assay involving counting expired larvae could serve as a preliminary test of water quality in underdeveloped areas without the need for costly laboratory techniques.

## Data Availability

All data from the present study are available by request from the corresponding author.
